# Well-free agglomeration and on-demand three-dimensional cell cluster formation using guided surface acoustic waves through a couplant layer

**DOI:** 10.1007/s10544-022-00617-z

**Published:** 2022-05-21

**Authors:** Jiyang Mei, Aditya Vasan, Uri Magaram, Kenjiro Takemura, Sreekanth H. Chalasani, James Friend

**Affiliations:** 1 Medically Advanced Devices Laboratory, Department of Mechanical and Aerospace Engineering, Jacobs School of Engineering and Department of Surgery, School of Medicine, University of California San Diego, 9500 Gilman Dr MC0411, La Jolla, San Diego, CA 92093 USA; 2 Molecular Neurobiology Laboratory, The Salk Institute for Biological Studies, 10010 N Torrey Pines Rd, La Jolla, San Diego, CA 92037 USA; 3 Department of Mechanical Engineering, Keio University, 3-14-1 Hiyoshi, Kouhoku-ku, Yokohama, Kanagawa 223-8522 Japan

**Keywords:** Surface acoustic wave, Acoustofluidics, Cell agglomerate

## Abstract

Three-dimensional cell agglomerates are broadly useful in tissue engineering and drug testing. We report a well-free method to form large (1.4-mm) multicellular clusters using 100-MHz surface acoustic waves (SAW) without direct contact with the media or cells. A fluid couplant is used to transform the SAW into acoustic streaming in the cell-laden media held in a petri dish. The couplant transmits longitudinal sound waves, forming a Lamb wave in the petri dish that, in turn, produces longitudinal sound in the media. Due to recirculation, human embryonic kidney (HEK293) cells in the dish are carried to the center of the coupling location, forming a cluster in less than 10 min. A few minutes later, these clusters may then be translated and merged to form large agglomerations, and even repeatedly folded to produce a roughly spherical shape of over 1.4 mm in diameter for incubation—without damaging the existing intercellular bonds. Calcium ion signaling through these clusters and confocal images of multiprotein junctional complexes suggest a continuous tissue construct: intercellular communication. They may be formed at will, and the method is feasibly useful for formation of numerous agglomerates in a single petri dish.

**Supplementary Information:**

The online version contains supplementary material available at 10.1007/s10544-022-00617-z.

## Introduction

Cell cultures, regenerative medicine, and tissue engineering rely on efficient production of cell agglomerates to replicate human body processes and functions for biological and clinical applications (Bao and Suresh 2003; Berthiaume et al. 2011). In 1907, Harrison established cell culturing to probe the origin of nerve fibers. His technique and subsequent improvements enabled the continuous observation of tissue growth and differentiation of targeted cells outside the body (Harrison et al. 1907). Cells are traditionally seeded and grown on a flat substrate, such as a flask or a petri dish, in which cell culture media and antibiotics are added to ensure cell health in a single layer at 37 $^\circ$C (Abbott 2003). Though monolayer cell cultures are used to probe signaling pathways, recent studies have demonstrated that these cultures behave much differently than tissue in *in vivo* physiological conditions. West (2017) Consequently, information obtained from two-dimensional (2D) cultures may be misleading in real tissues, particularly for intercelluar functions, *e.g.*, proteins in the matrix (Frantz et al. 2010), structural architecture (Pampaloni et al. 2007), and cell-to-cell interactions (Thiery 2003).

Three-dimensional (3D) cell cultures have been shown to overcome these limitations, better mimicking the *in vivo* complex microenvironment and intercelluar behaviors of animal tissues (Jacks and Weinberg 2002; Lancaster and Knoblich 2014). Moreover, these 3D systems have been used in biology and tissue engineering (Oh et al. 2017), drug screening (Tung et al. 2011), and tumor metastasis (Bersini et al. 2014), tumor angiogenesis (Chung et al. 2017), toxicology (Ramaiahgari et al. 2014), and cell proliferation studies (Mandal and Kundu 2009).

As agglomerated 3D cell cultures, organoids and spheroids simulate a live cell’s *in vivo* environmental conditions far better than two-dimensional cell cultures (West and Brown 2005), which makes them useful for tracking physiological changes (Fennema et al. 2013; Laschke and Menger 2017). Spheroids, an older construct, are 3D spherical clusters of cells with a necrotic core, formed mostly from cancer cell lines or tumor biopsies where the importance of cell-cell interactions and the morphology and behavior of cells in real tissue are important (Chen et al. 2019). Organoids, organ-like structures produced from small fragments of tissue in an extracellular scaffolding environment, were initially regarded as an extension of three-dimensional (3D) cultures. They are able to exhibit functionality similar to the organs from which they are derived (Fatehullah et al. 2016; Park et al. 2019). Placed between single cell-based evaluations and animal testing, multicellular constructs like these more closely match the oxygen, nutrient, and waste gradients observed in avascular tumors, beneficial for anti-tumor therapy (Vinci et al. 2012) and cancer drug development (Pampaloni et al. 2007). For example, self-organizing organotypic organoids established from stomach (Bartfeld et al. 2015), kidney (Takasato et al. 2014), liver (Takebe et al. 2013; Broutier et al. 2017), intestine (Spence et al. 2011), and breast cancer biopsies (Sachs et al. 2018) contribute to better, more physiologically relevant models of healthy and cancerous human tissue. And, very recently, brain organoids of Neanderthals have been devised to study how modern human brains evolved from this closely related, extinct species to make *homo sapiens* unique among the archaic hominids (Trujillo et al. 2021). Notably, larger organoids produce more organ-like behavior, and it is desirable to have organoids larger than 1 mm in diameter in many cases (Wan 2016).

However, it is difficult to fabricate uniform and high-quality 3D cell agglomerates to eventually form spheroids or organoids for screening and testing. Existing methods include hanging droplets (Tung et al. 2011; Timmins et al. 2004), matrices Kleinman and Martin (2005) and scaffolds (Dosh et al. 2017), self-formation on non-adherent surfaces (Napolitano et al. 2007), magnetic-assisted assembly (Souza et al. 2010), the forced-floating method (Ingram et al. 1997), and dielectrophoresis (lbrecht et al. 2006). These techniques individually suffer from multiple disadvantages. For instance, some are low throughput and tedious, while others require careful labeling of individual cell clusters. A majority of these methods depend upon complicated or specific equipment, like prepared magnetic particle-containing hydrogels or a dielectrophoretic chamber. Most importantly, none of these methods rapidly form cell agglomerates.

Acoustofluidic devices have become rather broadly accepted to manipulate biological matter without contact at the submillimeter scale, as comprehensively reviewed in the past (Connacher et al. 2018; Friend and Yeo 2011), serving to engineer tissues (Choi et al. 2007), sort cells (Zhang et al. 2020; Kurashina et al. 2017; Imashiro et al. 2020), diagnose clinical conditions (Zhou et al. 2014), probe intercellular signaling (Faley et al. 2008), deliver drugs (Mayol et al. 2014), and analyze single cells (Prakadan et al. 2017). These microfluidic devices have also been further applied to cell culturing, especially for 3D tissue constructs (Huang et al. 2011; van Duinen et al. 2015; Huh et al. 2011). Bulk acoustic waves (BAW) in particular have been reported to help in forming agglomerates, but it is difficult to maintain uniform results due to the complicated design of the resonator structure (Bruus 2012). Later studies have shown that biological specimens can be translated and focused under a pressure gradient created by surface acoustic waves (SAW), which is known to manipulate cells (Guo et al. 2015, 2016) without changing their characteristics (Li et al. 2009). Acoustic streaming is also commonly used to concentrate cells and apply drag forces via the surrounding flow (Kurashina et al. 2017). Despite all this effort, a need remains for a reliable, contact-free, flexible, label-free, and biocompatible approach to rapidly form 3D cell clusters.

**Fig. 1:**
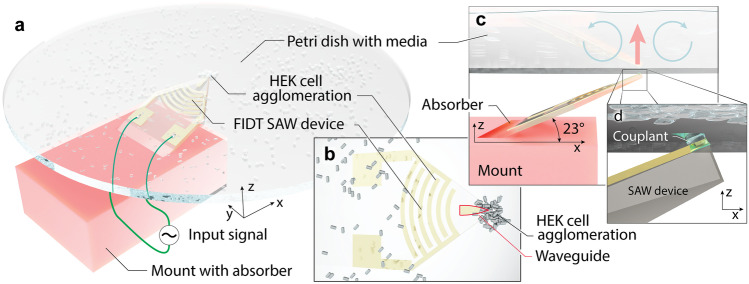
The cell agglomeration device. The **a** lithium niobate (LN) substrate is held in place by a mount with an absorber to prevent spurious and reflected acoustic waves at the Rayleigh angle of 23$^\circ$. This structure is mounted below a petri dish laden with media and HEK cells. Focusing surface acoustic waves generated by an input signal into a (**b**) focusing interdigital transducer (FIDT) on a lithium niobate substrate propagate into the superstrate (petri dish) through an Au focusing waveguide (thickness exaggerated, outlined in red) and into $<\,0.2$ $\mu$L Tween 20 as a couplant. The **b** view is from above and along the $-z$ axis through the media, cells, and petri dish. The mount is omitted for clarity. Acoustic energy is passed **c** vertically through the **d** couplant (along the *z* axis), through the petri dish, and into the cell-laden media (thick arrow). Acoustic streaming is generated in the media, in turn leading to a (**c**) local recirculation region (thin arrows) around the coupling position. This, in turn, leads to (**a**, **b**) cell agglomeration above the SAW device’s 40 $\mu$m wide tip. (Cells, Au thickness not to scale for clarity)

Here we propose an on-demand method for rapidly and controllably creating cell clusters in an open petri dish using SAW through a thin couplant layer. In our system, illustrated in Fig. 1, the SAW is generated from curved interdigital transducers (IDTs) that laterally focus it to a width of about two wavelengths on the substrate. By introducing a fluid atop the SAW-carrying substrate, this acoustic energy is converted—or *leaked*—into the fluid as longitudinally-propagating acoustic waves at the Rayleigh angle. By mounting the SAW device at this same angle, it is possible to vertically propagate the acoustic waves through the couplant fluid until they come into contact with a petri dish. Lamb waves (Hodgson et al. 2009) are produced from modal conversion in the dish substrate, in turn causing longitudinal acoustic waves to be formed in the cell-laden fluid within the petri dish. These acoustic waves propagate at a different Rayleigh angle, as the speed of the Lamb wave in the petri dish is not the same as the speed of the SAW across the lithium niobate (LN) substrate. Acoustic streaming induced by these propagating longitudinal acoustic waves generates local regions of fluid recirculation sufficient to accumulate cells into small clusters. Using this method, we demonstrate local multi-layer cell agglomeration within a well-free container. We further demonstrate the ability to *simultaneously* form several adjacent clusters in a cell culture dish with a set of SAW devices, and then to combine these clusters to form much larger 3D cell agglomerations than have been seen in past work. In what follows, we explain the details of this technique, illustrate its use, and combine imaging and analysis to illustrate how this technique may be used to easily produce 3D cell cultures on demand.

## Results and discussion

### Working mechanism

**Fig. 2:**
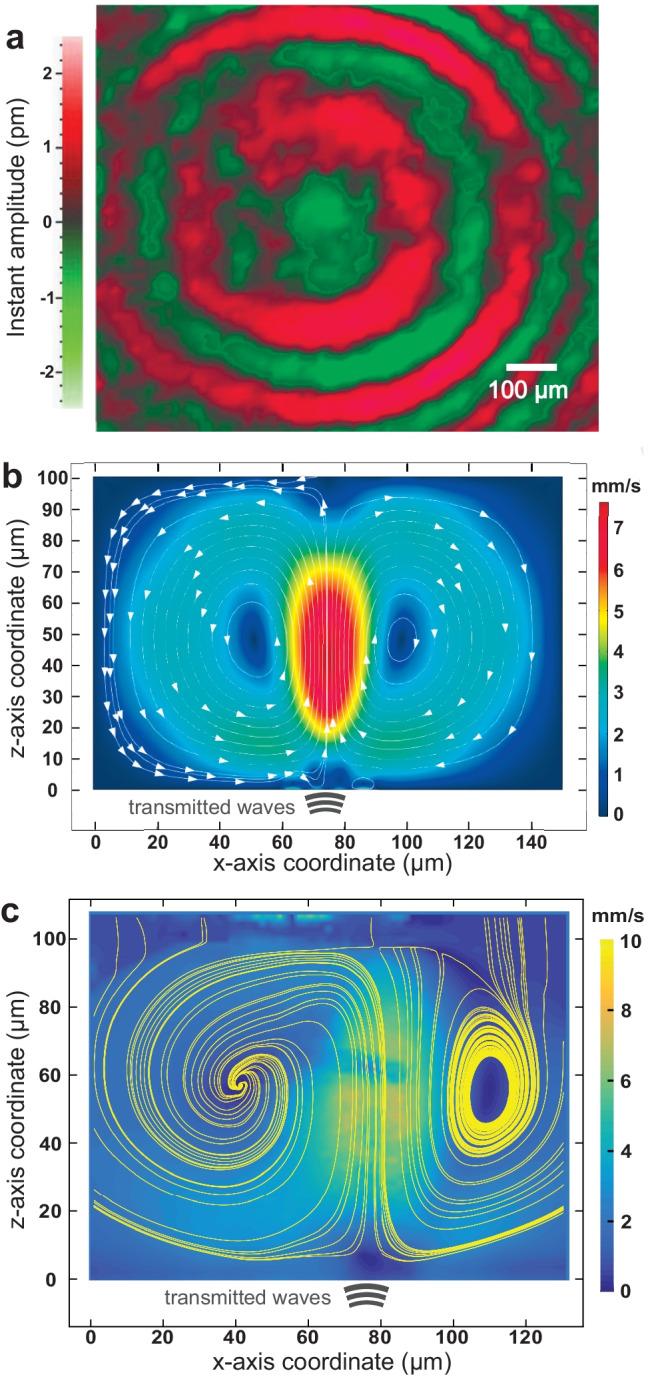
Generation of local vortical flow by leaky Lamb waves from the coupling point**.** **a** LDV measurements display the Lamb wave propagating along the petri dish, from roughly at the center, the source of acoustic energy from coupling with the SAW device from below. The Lamb wave concentrically spreads out from the center of transmission, where red and green colors denote the instantaneous peaks and valleys of the vibration. Scale bar: 100 $\mu$m. Side views of the recirculation in the fluid within the petri dish, as actuated by the transmitted acoustic waves from the SAW device through the couplant liquid, and onward through the glass of the petri dish into the fluid within, using **b** finite element analysis, and **c** experimental $\mu$PIV, where the background color represents the velocity magnitude and the yellow lines display the streamlines. The petri dish’s top surface is at $z=0$, and the couplant is centered at $x=78~\mu$m along the *x* axis

To actuate a small area, focused SAW was generated from the focused interdigital transducer (FIDT) on the lithium niobate substrate (see Fig. 1(a)). The fabrication and experimental details are described in the *Methods*. A thin 400-nm layer of gold was patterned in a triangular shape as a waveguide to overcome wave steering due to the anisotropic nature of the lithium niobate substrate and further confine the acoustic energy to the tip (Mei and Friend 2020). The tip of the waveguide was set to have a 40 $\mu$m width to match the wavelength of the SAW as the minimum possible width of the confined SAW in the LN substrate. From formation within the IDT, SAW was propagated on the substrate until it encountered the couplant liquid (Tween 20) placed at the waveguide tip. Upon propagating under the liquid, the SAW diffracted at the Rayleigh angle into the couplant liquid to produce sound. The Rayleigh angle is determined by the speed of sound in the two media, with $v_{\text {SAW}}$ as the speed of the Rayleigh SAW in LN and $v_\text {fluid}$ the longitudinal speed of sound in the coupling fluid, $\theta _R=\sin ^{-1} \left( v_{\text {fluid}}/v_{\text {SAW}}\right) =23^\circ$. As the SAW device was tilted at this angle, the longitudinal sound waves were transmitted vertically toward the superstrate, where they were converted to Lamb waves in the superstrate material. The superstrate was a petri dish in our study that contained cell-laden media (see Fig. 1(b)). Viewed from the top, the vibration induced in the petri dish propagates concentrically outward from the coupling location as shown in Fig. 2(a). The wavelength of this propagating wave, about 100 $\mu$m, is longer than the SAW in the LN device source, because the velocity of the Lamb wave in the petri dish is higher. The propagating leaky Lamb waves produced longitudinal sound waves in the fluid medium, leading to acoustic streaming sufficient to induce recirculation in the medium.

The power of the SAW, and therefore the velocity of the recirculation, was controlled such that the cells would be translated to the center by the drag force, but would not be pushed up and carried away by the recirculation, along the lines of past work using other devices (Kurashina et al. 2017). This produced a monolayer of cells adjacent the petri dish’s bottom surface. The hydrodynamic drag from the flow at low Reynolds number can be derived from Stokes’ equation as $F_d=6\pi \eta rv$, where $\eta$ is the dynamic viscosity of the medium; *r* is the radius of the particle; and *v* is the flow velocity. Later, we show that as the size of the “particle” grows through agglomeration of cells, it becomes possible to lift and fold these monolayer agglomerates to form large multilayer groupings of cells.

### Cell agglomeration

We first considered the ability to rapidly agglomerate cells using the device. As described in Sect. 4.2, HEK293 cells were diluted to a density of $1.25\times 10^5$ cells/mL and 1.2 mL of the suspension was transferred to the low-attachment petri dish. A recirculation vortex formed in the fluid contained by the petri dish during exposure to acoustic energy from our device. The flow carried unattached cells present in this vortex to the center of the actuation area, gradually forming a flat, monolayer to few-layer cluster. Power sufficient to initially move the cells was about 15 mW, although this is insufficient to draw the cells into the vortical flow. The velocity of the cells may be intrinsically adjusted by altering the power input. However, when the power was increased above 92 mW, individual cells were lifted into the recirculation and lost instead of adhering to the nascent agglomerate, greatly slowing the agglomerate’s growth. Compared with the hours to days required for agglomeration to form using other methods, our technique accumulated the cells together within minutes. Whatever the method, incubation is required for a few hours so that the cells are bound together by an actin network. In our method, this is fortunately accomplished without needing to apply SAW after forming the initial agglomerate.

**Fig. 3:**
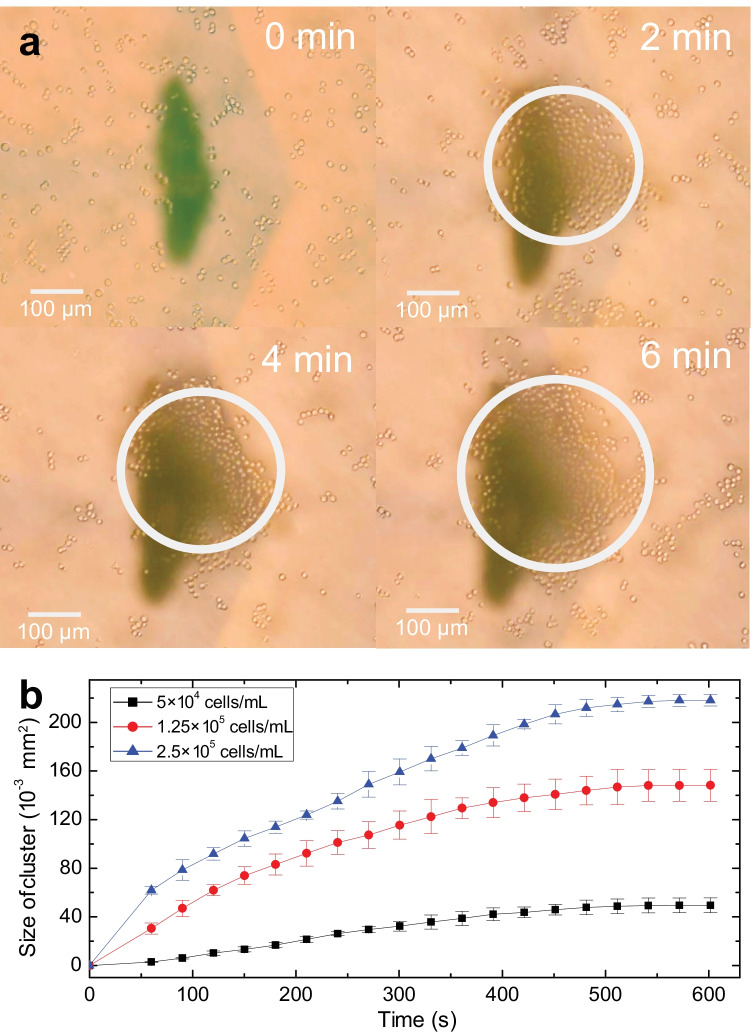
Cell agglomeration via coupled SAW-Lamb waves. Initially, cells are homogeneously distributed in the media at a concentration of $1.25\times 10^5$ cells/mL with no SAW. Upon activating 92 mW SAW in this arrangement, cells are gradually accumulated above the coupling point where the acoustic wave is transmitted from below, indicated in the images above with a dark right-handed chevron shape. The white circles outline the grouping of cells that gradually grow over time until they reach an almost steady state. This is more clearly shown by b) a plot of the cluster’s area with respect to time at various cell concentrations, $5\times 10^4$ cells/mL (black squares), $1.25\times 10^5$ cells/mL (red circles), $2.5\times 10^5$ cells/mL (blue triangles). After eight minutes, the cluster size reached a steady state in this experiment. The error bars denote the standard deviation from five measurements of the cluster size. A video of the phenomenon is provided in the Supplementary Information. Scale bar: 100 $\mu$m

Cells roughly agglomerate from a dispersed condition into a circular, flat monolayer to few-layer shape in 2 min with a diameter of about 280 $\mu$m, as shown in Fig. 3(a). The agglomeration grows with time to 310 $\mu$m at 4 min and 360 $\mu$m at 6 min. It is important to note that the dark, chevron-shaped spots in Fig. 3 were introduced to identify the point at which the couplant fluid touches the petri dish. The observed cell cluster area was measured and plotted in Fig. 3(b) for operation at 92 mW input into the SAW device. The error bars represent the standard deviation of the data, repeated five times per data point, with the average used as the main data point. Data were acquired from three parallel agglomeration experiments using the same batch of cells under the same power (92 mW).

The agglomeration’s growth slows over time, to essentially a constant at about 500 s or 8 min. Nearly all the cells have been extracted from the recirculating vortex by this time, and only rarely do cells from outside the vortex enter into it. Further, the size of the agglomerate with respect to time under the same input power at different cell density levels is also presented in Fig. 3(b), showing a similar trend. This indicates that the method works for different cell concentrations, though the final cluster size strongly depends on the concentration. In fact, the initial change in the cluster size with respect to time corresponds to the square of the cell concentration to less than 5% error. This is indicative of *orthokinetic coagulation*, a term coined long ago by von Smoluchowski (1917) to represent shear-based particle agglomeration (Shilton et al. 2008).

### Cluster translation

A natural extension from forming flat, monolayer to few-layer clusters of cells is to manipulate them. For example, moving them about in the petri dish without a pipette. The ability to form multilayer to wholly spherical agglomerations of cells would be even better. Moreover, if one wishes to increase the size of a cluster beyond what a given vortex recirculation cell can provide, it is reasonable to suppose the SAW device, the vortex cell it is generating, and the cluster entrapped within the cell could all be moved around to collect more cells and enlarge the cluster. Alternatively, one could create several clusters and merge them afterwards.

Similar to the process of agglomeration, where the cells are “dragged” by the flow into a quiescent point at the center of the vortical flow, translation of an entire monolayer agglomeration is also possible when the flow is sufficient. In our experiments, a cluster about 350 $\mu$m in size was translated over a 560 $\mu$m distance in 90 s at a speed of 5.77 $\mu$m/s using a power of 91.48 mW (see Fig. 4 and a video of the phenomenon in the Supplementary Information). In these results, the SAW device and microscope were held in a fixed position while the petri dish was moved. The cell agglomeration remained fixed in place with the SAW transducer’s tip while the petri dish was moved.

Unsurprisingly, the transport speed is reduced as the size of the object is increased. For example, a larger, elliptical, and thin cluster about 1029 $\mu$m $\times$ 750 $\mu$m in size was moved at about 2.35 $\mu$m/s. In doing so, it also shows how this cluster may be translated to come into contact with another, smaller 647 $\mu$m cluster. By placing the center of the induced flow vortex at a point between these two clusters, it was possible to merge them together to form a 1410 $\mu$m $\times$ 810 $\mu$m sized monolayer cluster. In this way it becomes possible to form rather large monolayer clusters of cells. To date, we have assembled monolayer clusters up to about 1510 $\mu$m $\times$ 1100 $\mu$m in size with this procedure. This size—and the size of the agglomerates we report later—is significantly larger than the majority of the existing work reported using conventional methods mentioned in Sect. 1, which are, at most, a few hundred micrometers in diameter. For example, intestinal organoids of about 40 $\mu$m have been formed by the hanging drop method (Panek et al. 2018), and adipose stem cell-based spheroids with a diameter of 80–110 $\mu$m have been formed using scaffolds (Zhang et al. 2015). These methods are the most popular among a broader variety of methods (Velasco et al. 2020). Moreover, the approach here produces larger cell agglomerations than other acoustic methods reported in the literature: Chen et al. (2019) reports a diameter of 300 $\mu$, Chen et al. (2016) reports a diameter of 200 $\mu$m, and Kurashina et al. (2017) reports a diameter of 70 $\mu$m. In the context of organoids, which are of recent interest, these sizes are generally smaller than the desired size of 1 mm or larger in most cases (Wan 2016).

The ability to define cell agglomerations that span a range from a few hundred micrometers to well over one millimeter may be beneficial. Larger 3D cultures can be expected to behave more like *in vivo* tissue, potentially exhibiting the cellular heterogeneity typical of solid tumors and tissue, where large gradients in nutritional and oxygen supply are responsible for different proliferation rates (Groebe and Mueller-Klieser 1996).

**Fig. 4:**
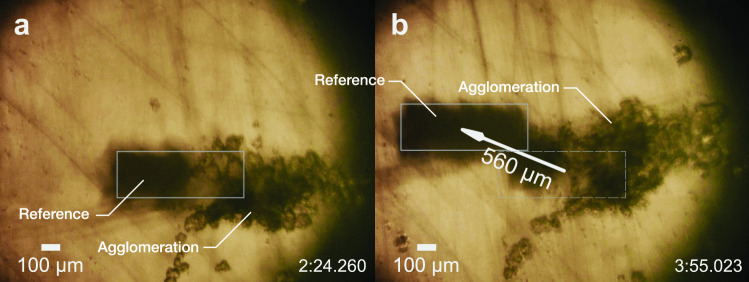
Translation of cell agglomerations in the petri dish. After agglomeration and waiting (with the transducer off) for about five minutes, the cell cluster may be transported along with the tip of the transducer underneath the petri dish. The (boxed) reference mark as shown is attached to the petri dish. By leaving the transducer coupling tip and the observation microscope fixed in place, and translating the dish up and to the left by 560 $\mu$m (for example) over a period of 90 s—from **a** 2 min 24.260 s to **b** 3 min 55.023 s—the agglomeration was moved downward and to the right by this distance relative to the petri dish. Scale bar: 100 $\mu$m

### Three-dimensional cellular agglomeration formation via origami-like manipulation

As previously mentioned, monolayer (2D) clusters have distinct drawbacks in emulating real tissue in comparison to 3D multilayer cell structures. To form these desirable multilayer agglomerations, the power must be increased so that the cells may be lifted from the petri dish’s surface and folded. It is possible to lift an edge of the thin cell agglomeration from the petri dish and lay it across the remaining layer of cells, all without breaking the intercellular connectivity defined in the original layer. This is analogous to origami folding used to produce micro to nanoscale devices from planar media (Rogers et al. 2016).

**Fig. 5:**
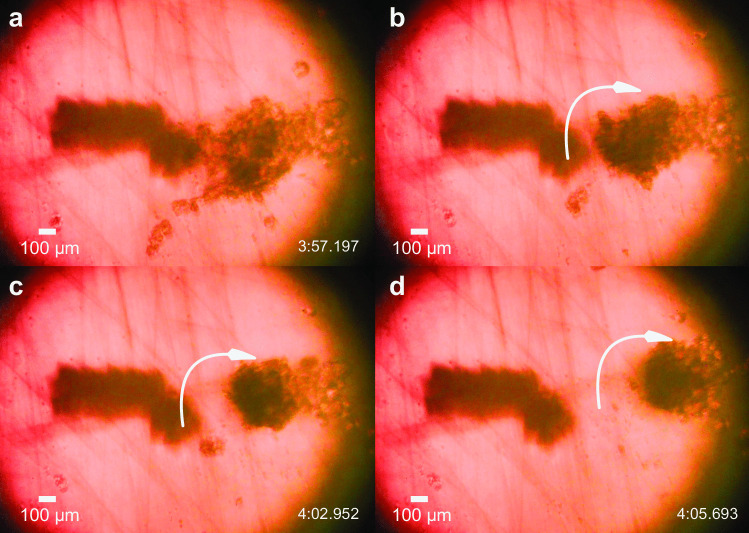
Cell agglomerate folding and rolling. After waiting for 5 min to weakly bind the existing cells together, increasing the input power to 350 mW causes the cluster to (**a**, **b**) roll upon itself from the left edge, folding atop the remainder of the cells and forming (c,d) a roughly spherical cell agglomeration in 8 s. Scale bar: 100 $\mu$m. Supplementary Video 2 shows the process in real time

Figure 5 illustrates an example of this folding procedure with our device. A five minute wait after forming the monolayer agglomeration is sufficient to produce some initial binding between the cells. At this point, it becomes possible to handle the agglomeration via pipetting or other tools as desired without fear of the agglomeration’s separation. Complete intercellular binding occurs only after several hours of incubation; the functional agglomerates we describe later were formed after 22 h incubation.

To have a sufficiently large number of cells in the agglomeration for a cluster, we adopted the following procedure after waiting for five minutes to allow for limited intercellular binding after SAW agglomeration. We exposed the left edge of the agglomeration to vortex flow generated from 350 mW input to the SAW device. The left side of the monolayer agglomeration folded up, over, and down upon the right side while the cells remained bound to each other. In this way, monolayer clusters can be folded from an arbitrary direction, defined by the location of the acoustic device’s coupling point with respect to the agglomeration’s edge. This folding process progresses to tumbling of the cell agglomeration, forming a compact and roughly spherical ball of cells.

At this point, one wonders how well the intercellular communication has developed among these agglomerated cells after their folding and subsequent 22-hour incubation. This is explored in the next subsection.

### Characterization of 3D cell agglomerations

Fluorescent imaging is vital to discern the quality of intercellular binding and communication to determine whether they exhibit cell-cell communication behavior or not. Clusters made with our device, first in a monolayer format and then folded to form a multilayer bound cluster, were carefully transferred with a pipette to an ordinary well plate for further observation. In order to ensure sufficient time for cells to bind together, they were incubated at 37$^\circ$C and 4.4% CO$_2$ for 22 hours before imaging; details are in the *Methods*.

#### Calcium migration

**Fig. 6:**
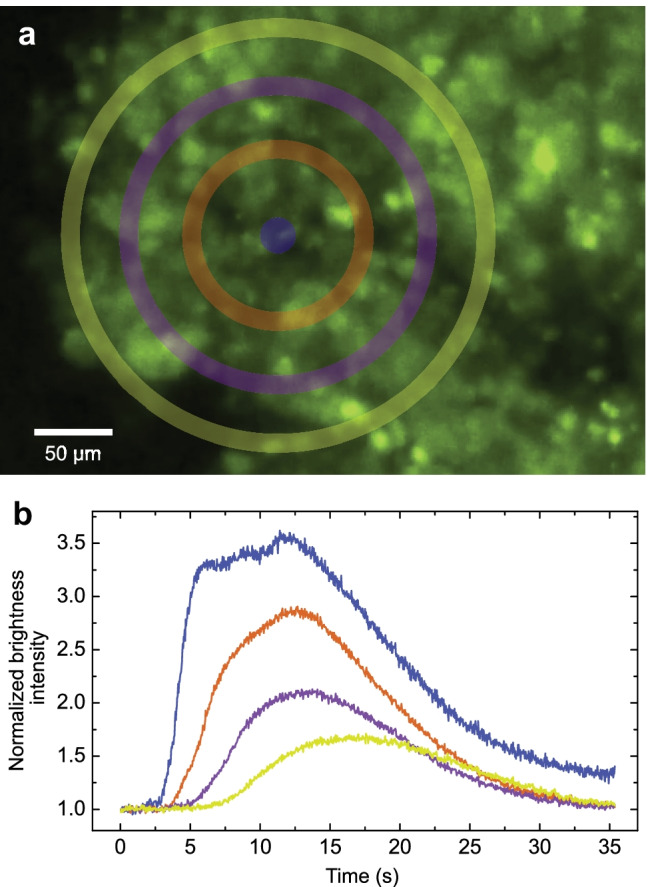
Calcium ion signaling in a cell agglomerate after 22 h incubation. The calcium (in green) transmission in a 3D cell agglomerate in the order from center to periphery (blue-orange-magenta-yellow). **a** The concentric regions of interest denoted on a photo of the cluster acquired from fluorescent microscopy. **b** The normalized brightness intensity mapping across of the corresponding regions. The Ca$^\text {2+}$ signaling seen here and in the video in the Supplementary Information indicates the cells are functioning as a collective group. Scale bar: 50 $\mu$m

Calcium (Ca$^\text {2+}$) (green in the images in Fig. 6—see Methods 4.6.1) is commonly used to identify communication among cells, as it plays a significant role in signal transduction through the cell membranes. Because the cells are nonuniformly distributed, calcium imaging is likewise nonuniform. However, intercelluar transmission of Ca$^\text {2+}$ signaling may be easily distinguished in the sequence of images and associated brightness in Fig. 6 and as a video in the Supplementary Information. The calcium signal emerged at the center (blue) in Fig. 6(a) and concentrically spread out (blue-orange-magenta-yellow) in about 16 s, showing a gradual progression and weakening of the signal from the source as time elapsed in Fig. 6(b).

#### Marking cell junctions

**Fig. 7:**
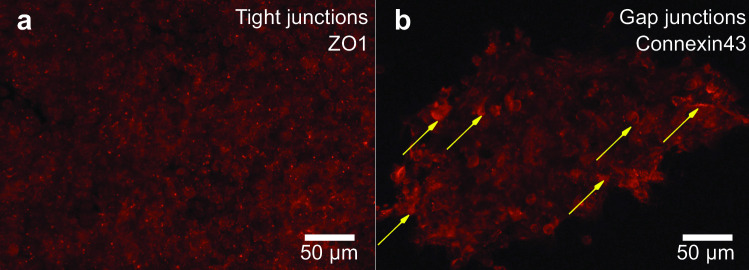
Indication of tight and gap junctions in the cell agglomeration. Confocal images of multiprotein junctional complexes (in red) in acoustically-formed 3D clusters. **a** Tight junctions in the cluster support intracellular bonding, maintaining the aggregated structure. **b** Gap junctions (stronger signals marked by yellow arrows) permit intercellular communication, including Ca$^\text {2+}$ propagation in the cluster as shown in Fig. 6. Scale bar: 50 $\mu$m

Cell morphology and intercellular contact are essential for defining and modulating cellular functions for *in vitro* cell cultures. Immunohistochemistry is widely used in basic research to identify the presence of certain proteins, and to understand the distribution and localization of biomarkers in different parts of a biological tissue. In order to better understand and explain the calcium propagation illustrated in Fig. 6, we evaluated the establishment of tight and gap junctions among adjacent cells in the SAW-formed clusters by staining ZO1 and connexin43 (see Methods 4.6.2). Images from confocal microscopy show the stained tight junctions and gap junctions, both in red. The ubiquitous presence of the tight junctions in Fig. 7(a) suggests the cells are bound together, as this is one of the functions of tight junctions. Fig. 7(b) indicates the gap junctions (marked by yellow arrows), which support the passage of various molecules, ions and electrical impulses between cells, potentially contributing to the previously discussed calcium propagation in Fig. 6. The presence of tight junctions and gap junctions along the cell membranes suggests the cells have formed tissue-like connections, revealing intercellular interaction behaviors from these agglomerated cells. The possibility exists to form organoids if the method is applied to stem cells.

**Fig. 8:**
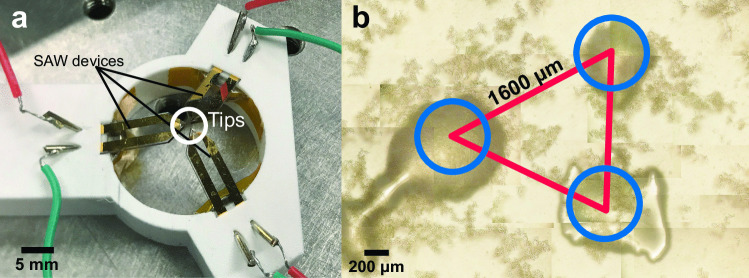
Simultaneous formation of multiple agglomerates. **a** The platform holding three mounted SAW devices could transmit waves to the superstrate at three distinct locations. **b** Three clusters, at sizes of about 500 $\mu$m, were simultaneously made in the petri dish

### Forming multiple cell clusters in a single petri dish

A key advantage of this method is the ability to make 3D-structured clusters on demand. The efficiency of agglomerate construction can also be increased by creating arrays of clusters in the same container, without—for example—having to resort to individual wells in a well plate. As shown in Fig. 8(a), a 3D-printed platform was designed so that three SAW devices could be inserted and actuated at the same time to simultaneously form three clusters.

The tips of the devices were adjusted to be on the same plane with a 1.8 mm lateral separation from tip to tip. The petri dish was set level above these three tips, with a gap of 350 $\mu$m. Each tip was loaded with about 0.15 $\mu$L of couplant to span this gap and to transmit the acoustic energy into the cell-laden medium through the bottom of the petri dish. In this way, the cells may be driven to accumulate at each of the three points due to localized recirculation as described in Sect. 2.2. In Fig. 8(b), the distances between each of the three cell agglomerations was about 1.6 mm. As the size of each agglomeration grows, it may become necessary to increase the separation distance to prevent them from merging. For example, with three 500-$\mu$m agglomerations, 1.6 mm is entirely sufficient. However, if each agglomeration grows beyond about 1 mm in size, they begin to interact and will merge.

A question then arises as to how close these three regions may be placed before it becomes impossible to collect cells to form agglomerations at each point. Here, with a cell concentration of $1.25\times 10^5$ cells/mL and 100-MHz SAW, the smallest separation possible is 711 $\mu$m, regardless of the cluster size. The SAW power used to obtain this result was 20.83 mW; other input powers required greater separation distances for individual clusters. This implies that, if the agglomerates are hexagonally close packed (Conway and Sloane 1998), and each agglomerate is sufficient by itself to use as a 3D cluster after folding it into a multilayer structure, it should be possible to form over ten thousand agglomerates in a 35-mm diameter petri dish like the one we used. Conservatively, at least a thousand agglomerates should be possible in a single petri dish.

Of course, the key limitation is not space, but time, in that the time required to achieve this with three SAW devices—almost exactly two weeks—would be greater than the viable life of the cells. However, if the idea were developed beyond the research context with a greater number of SAW devices, either monolithic (on the same substrate), stacked, or both, then the time to form so many clusters becomes more reasonable: a thousand such devices would be able to produce ten thousand agglomerates in about an hour. The cost for us to produce these devices today in our lab is about $50 each, yet in greater volumes there are many examples of SAW devices being produced at less than $1 each. Moreover, because the hydrodynamics is relatively slow compared to the acoustics, one can employ intermittent drive strategies such as pulsed width modulation (Rajapaksa et al. 2014) to sequentially operate the devices from a modest power supply. Finally, the use of intermittent drive strategies can mitigate concerns with heating; fortunately here the power required is low compared to other SAW applications (Rajapaksa et al. 2014) where heating can become important to consider.

Alternatively, one can imagine a flow-through device to accomplish the same outcome, but only by trading the complexity of so many SAW devices with the complexity of cluster handling in specialized microfluidic dishware. This is likely to be difficult during the time spent waiting for the cells to adhere to each other after agglomeration—and hopefully not the walls of the microchannels in the device.

**Table 1:** Changing the power controls the essential functions of the SAW-driven cell agglomeration and manipulation device in this study, from agglomeration to translation and folding of agglomerated cells

Function	Input $V_{pp}$ (V)	Input power (mW)
Agglomerate	3.01	14.32
	4.6	32.29
	6.01	56.40
	7.56	91.64
Translate	9.04	130.7
	10.83	227.6
Fold	15.06	357.4
	22.53	894.7

## Conclusions

Three-dimensional cell cultures are useful tissue analogs for biomedical research applications. To date, creating agglomerates is tedious with individual clusters formed in pendant droplets with the hanging drop method or individual, nonadherent-treated wells for the well-based method, both representing a physical separation that requires skill in handling and use. Through the use of coupled SAW with modal conversion into a Lamb wave (Hodgson et al. 2009), which transmits the acoustic energy through a simple petri dish, cells suspended in media in the petri dish may be agglomerated to form a cluster in less than ten minutes in a location of the user’s choice. In our case, we employed human embryonic kidney (HEK293) cells to form the agglomerations. After formation of the agglomerated cells, they remain bound together. To improve their suitability as clusters, we used our device once again to combine several of the individual agglomerates together and folded that result into an irregular but bound structure shown to transmit Ca$^\text {2+}$ signaling and gap and tight junctions among the cells. Table 1 indicates the input power required to perform agglomeration, translation, and folding. At power levels less than tabulated here, there is little to no cell motion. Beyond these power levels, recirculation is sufficient to prevent formation of any cell clusters, instead causing the cells to rapidly circulate above the contact point of the SAW device.

This work illustrates a potential method that, when developed further, could help the reader conveniently produce large numbers of organoids. It illustrates the potential of acoustofluidics in streamlining laboratory procedures through a simple chip device, even with standard laboratory dishware.

## Methods

### SAW device fabrication

A resonant frequency of 100 MHz for the SAW device was selected based on a desire to have a rapidly attenuating acoustic wave (Dentry et al. 2014) in the cell-laden media after it is converted from SAW to sound, a Lamb wave, and to sound again as it passes through and into the petri dish (Hodgson et al. 2009). This helps reduce reflection of the acoustic wave in the media, and, with the reduction in the wavelength as the frequency is increased, it also helps to facilitate easier manipulation of the individual cells to form an agglomeration. A circularly-focused IDT (FIDT) was deposited on a piezoelectric substrate (LN, 127.68$^\circ$ *y*-rotated, *x*-propagating, single crystal, double-side optically polished lithium niobate, PMOptics, Burlington, MA USA) with unweighted and equally spaced fingers to produce a wavelength of 40 $\mu$m ($\lambda =v/f$, where $\lambda$, *v*, and *f* are the wavelength, velocity, and frequency, respectively, of the SAW on the substrate). Details of the fabrication process are provided elsewhere (Mei et al. 2020), and a brief summary specific to these devices is given here. Standard ultraviolet (UV, 375 nm) photolithography was used for the fabrication of the device, using a negative photoresist (NR9–1500PY, Futurrex, NJ USA) and associated developer (RD6, Futurrex, NJ USA). This was followed by sputter deposition (Denton 18, Denton Vacuum, NJ USA) of 400 nm Au atop 5 nm Cr, with the latter as an adhesive layer. A triangle-shaped guiding layer with tip width at 40 $\mu$m was deposited at the same thickness using the same method to serve as a waveguide for the SAW and reduce lateral diffraction losses (Mei and Friend 2020). The SAW device was mounted on a 3D-printed platform at the Rayleigh angle (23$^\circ$).

### Cell culture

Human embryonic kidney cells (HEK293 cells, CRL–1573, ATCC (American Type Culture Collection), Manassas, VA USA) were cultured using standard procedure in Dulbecco’s Modified Eagle Medium (DMEM, MilliporeSigma, Burlington, MA USA) supplemented with $10\%$ fetal bovine serum (FBS, MilliporeSigma, Burlington, MA USA) and 20 mM glutamine in a $37~^{\circ }$C and 4.4% carbon dioxide (CO$_2$) incubator (Model 370 Steri-Cycle CO$_2$ Incubator, ThermoFisher Scientific, Waltham, MA USA). Cells beyond passage thirty were discarded in favor of a lower passage aliquot. Cells were trypsinated in the native container and triturated before being moved into an ultra-low attachment 35 mm petri dish (MS-90350Z, S-bio, Hudson, NH USA) for subsequent cluster formation.

### Actuation and measurements

The SAW was powered using a sinusoidal signal input using a signal generator (WF1967 multifunction generator, NF Corporation, Yokohama, Japan) and amplifier (5U1000, AR Instrumentation, Souderton, PA USA). The voltage and current were monitored to allow for the power to be calculated by the oscilloscope (InfiniVision 2000 X-Series, Keysight Technologies, Santa Rosa, CA USA). A small droplet ($\le 0.2$ $\mu$L) of wetting, surfactant-laden couplant liquid, (Tween 20, #9005-64-5, Cole-Parmer, Vernon Hills, IL USA), was introduced between the mounted lithium niobate substrate and ultra-low attachment petri dish superstrate. The fluid choice essentially eliminated fluid loss to evaporation and ensured wetting of both surfaces. The surface vibration was measured by a laser Doppler vibrometer (UHF–120SV, Polytec, Irvine CA USA and Waldbronn, Germany). All images and videos were acquired by a digital single lens reflex camera (D5300, Nikon, Minato, Tokyo, Japan) attached to a long-working distance microscope (K2-DistaMax, Infinity Photo Optical, Centennial, CO USA) with a 5X objective lens (Mitutoyo M Plan Apo 5X LWD Objective, Edmund Optics Inc., Barrington, NJ USA).

### Simulation of fluid flow due to SAW

We simulated the acoustic field due to surface acoustic waves propagating into the medium using finite element analysis (COMSOL Multiphysics 5.5, Comsol Inc., Los Angeles, CA USA) following an approach similar to the one used by (Nama et al. 2015) This involved using a perturbation approach that resulted in first and second-order equations for the acoustic radiation force and acoustic streaming behavior, respectively, which were successively solved. The second-order results were time-averaged to determine the acoustic streaming-driven fluid velocity. The resulting flow field due to streaming shows an upwelling region at the center, surrounded by a toroidal vortex with inflows along the bottom. This is similar to the experimentally observed flowfield using particle imaging velocimetry (PIV), as shown in Fig. 2. The simulation domain was modeled to replicate the geometry of the petri dish with fluid with the 100-MHz transducer at the bottom of the cavity. The left, right and top boundaries of the domain were defined to be walls, to correspond to the geometry of the petri dish. The magnitude of displacement of the transducer face was measured via laser Doppler vibrometry (LDV) to be about 1 nm in amplitude in these experiments, a velocity boundary condition was used for the transducer surface instead. The mesh size was defined to be one-sixth the viscous penetration depth ($\delta _v$/6; consult the Supplementary Information for a sample of the mesh) at the frequency of operation close to the walls. The fluid in the domain was assumed to have the properties of water.

### Visualization of fluid flow using $\mu$PIV

In order to visualize and experimentally simulate the trajectories of particles suspended in the SAW-driven recirculation within the petri dish, micro-particle image velocimetry ($\mu$PIV) was used. Polystyrene fluorescent particles ($\phi =43.2~\mu$m, #18242-2, Polysciences Inc., Warrington, PA USA) were introduced into a 35 mm petri dish filled with water, mimicking cells in medium in the dish. The particles were illuminated at 455 nm (M455L3, ThorLabs, Newton, NJ USA). When the SAW was turned on and the recirculation established, the videos were acquired by a high-speed camera (Fastcam Mini UX100, Photron, San Diego, CA USA) at fifty frames per second via epifluorescence filtering centered at 455 nm and a long working distance microscope with a 5X objective lens described above, after which each frame of the videos was extracted to form image sequences (ImageJ 1.53g, National Institutes of Health, Gaithersburg, MD USA). The motion of the particles was then analyzed and calculated by PIVlab (Thielicke and Stamhuis 2014), revealing the velocity vectors and streamlines.

### Cluster characterization

#### Calcium migration

Ultrasound-formed clusters were analyzed using calcium imaging via an inverted microscope (IN480TC-HD18-HDM, AmScope, Irvine, CA USA). Prior to ultrasound cluster formation, a HEK293 cell line expressing GCaMP6f was generated using a GCaMP6f lentivirus (PLV-10181-50, Cellomics Technology, Halethorpe, MD USA) followed by fluorescence-activated cell sorting (FACS) to maintain 100% GCaMP6f positive cells. Clusters generated by ultrasound were left to rest in a 37$^\circ$C incubator for 22 hours and then moved to a 60 mm petri dish for imaging. To quantify the intensity and illustrate how the Ca$^\text {2+}$ concentration evolved across the imaged cells, a circular shape was picked as the region of interest (ROI) where the signaling (green) started to emerge. To measure the progressive signal propagation radially outwards, a series of concentric donut-shaped ROIs was identified and quantified. The pixel intensity was determined (ImageJ) for each region over the time frame and normalized to its own baseline fluorescence, which in this case was the first twenty frames of the video, so that corresponding brightness curves started at one and could be compared, in intensity and time-course.

#### Immunohistochemistry and imaging

Ultrasound-generated HEK293 cell clusters were fixed in 4% paraformaldehyde in phosphate buffered saline (PBS) for 20 minutes. Immunohistological stainings for tight- and gap-junctions were performed with ZO1 (abcam216880; 1:500, Abcam, Cambridge, UK) and connexin 43 (abcam11370; 1:500, Abcam, Cambridge, UK), respectively, followed by mounting of the clusters between a slide and a coverslip before imaging on a confocal microscope (LSM 800, Zeiss, Oberkochen, Germany) with a 20x objective scanning along the z-axis to visualize cross sections. Junction markers were imaged at 561 nm.

## Supplementary Information

Below is the link to the electronic supplementary material.

Supplementary file1 (MP4 8.67 MB)

Supplementary file2 (MP4 1.62 MB)
